# “Wow! It’s Cool”: How Brand Coolness Affects the Customer Psychological Well-Being Through Brand Love and Brand Engagement

**DOI:** 10.3389/fpsyg.2022.923870

**Published:** 2022-06-28

**Authors:** Saman Attiq, Abu Bakar Abdul Hamid, Munnawar Naz Khokhar, Hassan Jalil Shah, Amna Shahzad

**Affiliations:** ^1^Air University School of Management, Air University, Islamabad, Pakistan; ^2^PUTRA Business School, Universiti Putra Malaysia, UPM Serdang, Seri Kembangan, Malaysia; ^3^Department of Management Sciences, COMSATS University, Islamabad, Pakistan; ^4^NUST Institute of Peace and Conflict Studies (NIPCONS), National University of Science and Technology, Islamabad, Pakistan; ^5^AM MCR Ltd., Manchester, United Kingdom

**Keywords:** brand coolness, brand love, brand engagement, consumer well-being, customer delight

## Abstract

In this era of razor-edge competition, marketers strive to outperform their rivals by improving their brands. Increasing brand coolness may be the best way to do it. This study used a stimulus organism response (SOR) model by integration with brand attribution theory to conduct a cross sectional study using purposive sampling technique and surveying young consumers of smart gadgets in Pakistan. A total of 1,178 responses were received and analyzed by structural equation modeling. The results found a positive impact of brand coolness (stimulus) on brand love and brand engagement (both modeled as organism). Brand experience moderated these links. Brand love and brand engagement also mediated the relationship between brand coolness and consumer well-being and delight (both modeled as response). The findings suggest a very important contribution to theory and practice by testing unexploited outcomes of brand coolness. Especially, this study contributes to the consumer well-being literature, again an unexploited aspect of marketing literature. Despite the uniqueness of the findings, the cross sectional design of this study remains a major limitation. Future research may supplement the findings with the help of longitudinal studies. Marketers and practitioners may benefit from this study by improving the coolness of their brands so they may not only increase consumer engagement with the brand but they will also make consumers happy with their brands.

## Introduction

The extant literature has always focused on consumer satisfaction as an ultimate objective of marketing function. However, recent scholarship argues that market forces such as enhanced competition, technological changes, globalization, and evolving customer expectations have changed the landscape of consumer satisfaction (Wilder et al., 2014). Over a period of time, both researchers and practitioners have understood the fact that satisfaction, although important, is not enough to garner customer long-term loyalty and repurchase behaviors (Pansari and Kumar, 2017). Thus, “the goal of organizations has evolved from relationship marketing to engaging customers in all possible ways” (Pansari and Kumar, 2017; Rosado-Pinto and Loureiro, 2020). The field of marketing has also progressed much in terms of managing customers. For example, traditional transactional approach was adopted, followed by relational approach in which developing a long-term profitable relationship is a prime focus. Now, the current era is said to be the era of customer engagement (Pansari and Kumar, 2017). This approach, i.e., customer engagement approach, is being considered very important for attaining a competitive edge (Kumar and Pansari, 2016; Alvarez-Milán et al., 2018), as it has a positive influence over relevant attitudes and behaviors such as satisfaction (Brodie et al., 2013), loyalty (Hollebeek, 2011), and brand usage (Hollebeek, 2011). Resultantly, practitioners are emphasizing something beyond mere satisfaction and consider customer delight as its alternative.

The ultimate purpose of human activity has always been to achieve great happiness. Numerous economists, psychologists, and sociologists, among others, have emphasized the significance of understanding human happiness or well-being (Diener and Seligman, 2002). In the past, marketing research has focused on achieving consumer joy, building customer loyalty (Netemeyer et al., 2004), and motivating consumers to repurchase and re-adopt (Hellier et al., 2003), whereas recent studies on consumer behavior and brand consumer relationships has begun to focus on consumer well-being (Mogilner et al., 2012). The concept of customer well-being influences not only brand loyalty but also how consumers choose products and spread word of mouth (Schnebelen and Bruhn, 2016). Researchers have also looked at what makes consumers happy at different stages of their consumption (El Hedhli et al., 2016).

In brand management literature, certain researchers focused on how brand consumer relationship quality, attachment, and self-congruence with a brand relate with consumer well-being (Schnebelen and Bruhn, 2018). Past research on brand-consumer relationship literature has focused on variables such as brand love, referred to as consumers’ strong positive feelings for a particular brand (Carroll and Ahuvia, 2006). Marketing literature outlines the importance of brand love because of its strongly established empirical associations with other relatively favorable outcomes, i.e., loyalty (Bairrada et al., 2018; Coelho et al., 2019), word of mouth (Bairrada et al., 2018; Coelho et al., 2019), willingness to pay more and self-disclosure (Bairrada et al., 2018), active engagement (Bergkvist and Bech-Larsen, 2010), and well-being (Junaid et al., 2020).

This entails understanding how brands promote customer wellness by addressing their emotional and psychological requirements in the sphere of brand consumer interaction. Consumers nowadays are aware that brands have the ability to convey and elicit specific emotions (Isaksen and Roper, 2016). Users of Nike clothing, i.e., want to show their coolness, while Apple smartphone users want to communicate they are tech-savvy (Chaplin et al., 2014). This customer fit-in technique is critical in determining consumer brand choices (Albrecht et al., 2017). Brands are, therefore, focusing on differentiating factors to position their products. Technology-oriented products are highly standardized, having minor or no differences. These differences are not easily identifiable by a lay person. Sometimes, understanding minute differences need extra technical skills that many consumers do not have or do not bother about (Melewar and Lim, 2010). One way to differentiate between technology products is their “coolness,” i.e., a prime factor for consumer evaluation of products (Sundar et al., 2014). In simple words, brand coolness refers to the consumer’s perceptions of brands being aseptically artistic, appealing, reliable, useable, and authentic, and having appeal and higher status. Coolness in technology products is also considered to be an important factor for firms’ competitive edge, achievement of objectives, and product differentiation.

Research studies suggest that cool products such as iMac, iPad, iPhone, and iPod have made much growth in terms of sales and have transformed their parent companies into fortune-makers (Im et al., 2015). Young consumers are heavily dependent on smart gadgets. However, smart gadgets are considered to be difficult to differentiate because of the use of similar technology and higher level of resemblance between competing products (Ebrahim et al., 2016; Tiwari et al., 2021). However, the construct of product coolness is still at the stage of infancy and needs further exploration.

The outcomes of the current study are supposed to highlight the important role brand coolness can play in developing brand love and engagement in technology-related products. Moreover, the study will also highlight the mechanism through which distant outcomes such as customer delight and psychological well-being are achieved. The study also emphasizes the fact that in this era of intense competition, technology products need to attain customer delight through brand coolness that is focused on their branding strategies.

### Research Gap

The review of prior literature on brand characteristics has uncovered several open research gaps (i.e., theoretical, methodological, and contextual perspectives).

#### Theoretical Significance

Research on brand coolness is scarce and inconclusive; therefore, this study offers two major objectives: underpinning the concept of brand coolness in the technology-related context and understanding various components of brand coolness and their relationship to other brand-related emotional constructs (such as brand love and brand engagement) and outcomes (consumer well-being and customer delight). Moreover, consumer brand coolness and brand love have been acknowledged as one of the best ways for addressing the engagement of consumers. Previously, the major research stream regarding brand coolness has concentrated on cognitive variables like satisfaction (Liu and Mattila, 2019) and purchase intention (Liu et al., 2021). However, there was less focus on non-cognitive variables such as emotions, brand love (Tiwari et al., 2021), emotional arousal (Apaolaza et al., 2021), and passionate desire (Loureiro et al., 2020) despite the fact that emotions have greater influence over engagement behavior (Tiwari et al., 2021). Second, research on brand characteristics has outlined its impacts on various constructs such as brand love (Tiwari et al., 2021). Various studies have discovered significant relationships using the perspectives of “brand love” and “brand engagement” such as trust with satisfaction (Amegbe et al., 2021), image, symbolic personality, and brand hate (Kashif et al., 2021; Rodrigues and Brandão, 2021), social media involvement (Gómez et al., 2019), gamification (Xi and Hamari, 2021). In the same perspective, the contribution of other emotions and their processes on brand-related determinants of brand engagement such as brand coolness is also needed. The current study deals with understanding various components of brand coolness and their relationship with other brand-related strong emotional constructs (such as brand love and brand engagement) and outcomes (consumer well-being and customer delight). Third, researchers argue that there are various determinants that can predict the relationship between brand coolness and its brand-related emotional outcomes. Researchers (Kumar et al., 2021) have also emphasized the use of the stimulus-organism-response (S-O-R) theory to see how brand-related stimuli affect the organism to predict response. However, there is need to explore the mechanism through which this relationship can be understood and to test how characteristics of brands affect consumer emotions and behaviors. On the other side, the S-O-R framework has also been analyzed from different theoretical perspectives such as the triangulation theory of love (Kumar et al., 2021), brand relationship theory (Junaid et al., 2020; Kostritsa et al., 2020; Laato et al., 2020), and attachment theory (Koo and Kim, 2013; Mostafa and Kasamani, 2020). Therefore, understanding the mechanism of brand love using S-O-R, where O is brand love, can be of immense significance, especially when viewed in the background of the fact that the literature has mostly considered brand love as “response” in the S-O-R framework. Some other studies have looked at brand love as a sign of how people feel (Ali et al., 2021; Kumar et al., 2021). The current study uses different perspective to analyze S-O-R along with attachment theory. Therefore, there is a need to include a strong positive emotion-related aspect to investigate the limitations and attain deeper understanding of brand coolness to consumer well-being. This study, thus, tests brand love and brand engagement as “organisms” between brand coolness and its responses.

#### Methodological Significance

The existing research on brand coolness is at the developing stage; thus, the research has mainly focused on qualitative investigations to explore various issues related to its conceptualization and brand-related outcomes. A major limitation to qualitative studies on brand coolness is that these are unable to establish causality among the studied variables (Tiwari et al., 2021). Moreover, most qualitative studies on brand coolness have used smaller sample, thus creating desirability bias and threat to generalizability. The extant literature has identified from a single construct (Dar-Nimrod et al., 2012) that different studies use different components of “perceived coolness”(Sundar et al., 2014; Warren and Campbell, 2014; Bruun et al., 2016; Raptis et al., 2017), which limit our understanding of *perceived coolness*. Although some studies have shed light on the conceptualization of perceived coolness (Rahman, 2013), there is scarcity of empirical research on non-logical network of brand coolness (Chen and Chou, 2019; Cha, 2020; Loureiro et al., 2020; Apaolaza et al., 2021; Liu et al., 2021; Tiwari et al., 2021). With these limitations in the background, the current study examines the brand coolness concept with a quantitative method.

#### Contextual Significance

Research on brand coolness is overwhelmed by studies from the West, especially the United States and United Kingdom. However, consumers from different cultures may perceive brand coolness differently (Gerber and Geiman, 2012), and research from developing economies is scarce. This study, thus, focuses on consumers of a developing country especially, users of technology-related smart devices where brand coolness may work as a stimuli to affect positive emotions of brand engagement and brand love (both modeled as organism) and ultimately generate distant responses such as well-being and delight.

Finally, this study, therefore, aims at using brand personality concept such as brand coolness to estimate the consumers’ attitudes, emotions, and behaviors. In this context, this study intends to test how brand coolness correlates with brand engagement and brand love and, further, the effect of brand love and brand engagement on customer psychological well-being and customer delight, and the moderating effect of brand experience will be examined for technology products. The expected outcomes of this study will be significant, as recent researchers suggest the empirical testing of brand coolness (Tiwari et al., 2021) and its impact on different types of attitudinal and behavioral outcomes. Furthermore, researchers (Khan et al., 2021; Tiwari et al., 2021; Joshi and Garg, 2022) have suggested testing of brand love with other marketing variables, e.g., well-being (Junaid et al., 2020) and delight (Junaid et al., 2020). Moreover, researchers (Joshi and Garg, 2022) have suggested testing of the moderating effect of brand experience on other marketing constructs with different product categories (e.g., technology products). In simple words, this study aims to achieve the following objectives:

1. To examine the impact of brand coolness on (as stimulus) brand love and brand engagement (as organism).
2. To analyze the impact of brand love and brand engagement (as organism) on consumer delight and well-being (as response).
3. To evaluate the impact of brand experience on the relationship among brand coolness, brand love, and brand engagement.

The next sections will provide relevant literature, followed by methodology for data collection. The results of data analysis will be presented next, followed by discussion and recommendations.

## Literature Review

### Theoretical Foundation

This study used the stimulus-organism-response (S-O-R) framework to explain its framework (Russell and Mehrabian, 1974). The S-O-R framework is a popular model used to understand the response of organisms based on certain contextual and psychological stimuli (Laato et al., 2020; Kumar et al., 2021). This study likewise employs the SOR model to describe its research framework. This cognitive psychology-based paradigm gives a stronger foundation for comprehending consumer behavior. Consumer behavior is a function of an external stimulus and internal processing according to the basic tenets of this theory (Mehrabian and Russell, 1974; Jacoby, 2002). External stimuli, in combination with internal and psychological factors, influence behavior. This model has been widely utilized in environmental psychology, in which specific aspects of the environment serve as a stimulus (S), influencing a person’s internal feelings (O) and ultimately leading to a behavior (R) (Eroglu et al., 2001). However, the research has explored individual factors related to health and the environment while using the S-O-R framework (Kumar et al., 2021). The current study has conceptualized brand coolness as a stimulus in the area of technology products. This model contains three major components in which instead of a direct link between stimulus and response, first the stimulus links with the organism, and then the affective reactions of the organism are linked with behavioral response. In the framework of this study, coolness is modeled as a stimulus, brand love and brand engagement are measured as the organism, and response is modeled through consumer well-being and customer delight.

### Brand Coolness

Nowadays, coolness has been deeply studied in several areas, i.e., marketing (Warren et al., 2019; Loureiro et al., 2020), anthropology (Dar-Nimrod et al., 2012), psychology, and sociology, and characteristics of people and things have been mainly studied by coolness (Dar-Nimrod et al., 2018; Warren et al., 2019). Coolness had also been considered in the title of its origins (Nancarrow et al., 2002), vernacular usage, attributes (Rahman, 2013), elements of culture, characteristics of personality, and features of goods (Sundar et al., 2014; Bruun et al., 2016). The term “cool” was coined in 1960s in discussing the subculture which is the capital of cultural youth, arising in the cultural counter of black Americans. First, coolness is the abstract idea or it is the attribute of consumers (Belk et al., 2010). Second, coolness can also be an assessment of an individual. Productive consumers with ordinary backgrounds and their interests tend to differ about coolness of brands (Leland, 2004). Third, coolness features are highly dynamic and rapidly change with time (Wooten and Mourey, 2013). Fourth, coolness aids in the achievement of opposing ideas (Wooten and Mourey, 2013). Fifth, defining a product as cool reflects positive attributes of the product (Bird and Tapp, 2008). Sixth, coolness is a desirable trait that reflects social standing and association with cool communities (Horton et al., 2012).

Previous studies used brand coolness with multiple dimensions (Runyan et al., 2013; Sundar et al., 2014; Loureiro et al., 2020). To enhance the previous knowledge, the current study used five dimensions of coolness for technical products as recommended by authorities (Tiwari et al., 2021). They explored the coolness perceptions for the wearing gadgets interaction and they also suggested the structure of five elements of coolness along with the perceived use-ableness, innovativeness, attractiveness, usefulness, and attract sub-culture. Hence, contemplating smart gadget characteristics, there are five dimensions of this study (i.e., usability, reliability, originality, high status, and personal cool) to study the brand coolness construct and its impact on other attitudinal and behavioral responses.

#### Dimensions of Brand Coolness

Based on extensive literature review in the context of technology gadgets, the current study used five more relevant dimensions regarding brand coolness. The first element is reliability; it can be best defined as the ability to keep a promise and do correct things. Brands that have unreliable quality cannot attain brand coolness overtime. One popular case of Samsung is quoted in this regard such that when the Samsung model Galaxy S7 lost its reputation because of being unreliable. Its reputation score dropped by seven points in 1 year from 49 in 2015 to 42 in 2016. The second element is usability or usefulness; it is defined as the ability of a technology-related product to help users in performing intended tasks and enhancing performance. In technology products, usability is considered as an important aspect of coolness (Levy, 2006; Sundar et al., 2014). In 2017, a council presented a list of top 10 cool brands that consisted of PlayStation and Bose; the products were also frequently highly graded on the dimension of usability. In addition, usability has an impact on consumer intention to engage in product consumption for a longer period (Nascimento et al., 2018; Tiwari et al., 2021). Personal cool is the term rooted in self-concept theory that includes constructs concerned with young consumers’ perception of coolness of brands. “Self-concept denotes the totality of the individual’s thoughts and feelings having reference to himself as an object” (Sirgy, 1982). People tend to compare features of brands with their own self-concept such that they adopt brands that have congruence with self-concept. Having congruent brands adds symbolic meaning to user self-concept (Mehta, 1999). Originality is defined as “the level to which individuals think that a technological device is practically and aesthetically distinctive from the devices used in similar environments” (Kim et al., 2015). It is believed that devices whose external appearance is well-designed have superior functions and that interfaces that are easy to comprehend are considered distinct or original (Suh and Chang, 2006). Consumers having these devices feel themselves to be privileged and different from others who have traditional devices with less differentiating features (Kim et al., 2015).

#### Brand Coolness With Brand Love and Brand Engagement

The extant literature has documented a variety of outcomes of brand coolness. For example, Shin (2017), who found quality as an outcome of coolness (Im et al., 2015), suggested that brand coolness predicts perceived value, and Chen and Chou (2019) found a positive impact of brand coolness on attachment and loyalty. Some other outcomes include attitude (Warren et al., 2019), intention to use, and satisfaction (Liu and Mattila, 2019). The literature suggests brand love and brand engagement as a reliable means (Huang, 2019) of achieving consumer well-being (Junaid et al., 2020) and brand loyalty (Batra et al., 2012). Therefore, in this study, we examine the relationship of brand coolness with brand love and brand engagement. Furthermore, a plethora of outcomes about coolness of brands has been found by researchers. Tiwari et al. (2021) argued that perceived coolness is one of the powerful predictors of brand love for technology products. Therefore, the literature review suggests scarcity of studies that outline the brand-related outcomes of brand coolness such as brand love and brand engagement. Based on the literature, we propose the following hypotheses:

H1a: Brand coolness has a positive impact on brand love.
H1b: Brand coolness has a positive impact on brand engagement.

### Brand Love

The term “brand love” is derived from the psychological literature on love in interpersonal relationships (Batra et al., 2012). Love is a feeling that is intensely positive and is considered as an emotional attachment that is not equivalent to mere liking of brand (Carroll and Ahuvia, 2006; Rossiter, 2012). Brand love is “a higher-order construct including multiple cognitions, emotions, and behaviors, which consumers organize into a mental prototype” (Batra et al., 2012). Brand love can be defined as the “degree of passionate emotional attachment a satisfied consumer has for a particular trade name” (Carroll and Ahuvia, 2006). The consumer-brand relationship theory explains consumer brand love (Ferreira et al., 2019). This theory suggests that the existence of a relationship between a consumer and a brand is not enough, and that the quality, direction, and strength of the relationship matter most (Fournier, 1998).

#### Brand Love With Customer Delight, Psychological Well-being, and Brand Engagement

Brand love reflects positive feelings for an object, although recent research has found some variables that predict brand love such as brand trust, brand image, and brand satisfaction (Joshi and Garg, 2021). Brand love has been found to have an impact on brand engagement by other researches such as (Rodrigues and Brandão, 2021; Tran et al., 2021) and the connection of consumer brand (Tran et al., 2021), perceived coolness (Tiwari et al., 2021). However, researchers have found several outcomes of brand love such as word of mouth (Amaro et al., 2020; Rodrigues and Brandão, 2021), brand defense (Ali et al., 2021), brand hate (Kashif et al., 2021), brand engagement (Junaid et al., 2019; Joshi and Garg, 2021), and revisit intention (Amaro et al., 2020). Some other researchers found that brand love have a positive relationship with brand loyalty (Bairrada et al., 2018; Salem et al., 2019; Khan et al., 2021), overall brand equity (Verma, 2021), and consumer well-being (Junaid et al., 2019, 2020). In the same way, researchers found different emotional factors that affect customer delight, i.e., joy (Ball and Barnes, 2017; Barnes and Krallman, 2019), surprise (Ball and Barnes, 2017; Barnes and Krallman, 2019; Torres et al., 2020), and cognitive and affective experiences (Lee and Park, 2019). This study considers brand love as an emotional construct that, like other variables, may affect customer delight and psychological well-being. Based on the given literature, the following hypotheses are developed:

H2a: Brand love has a positive impact on customer delight.
H2b: Brand love has a positive impact on consumer’s psychological well-being.
H2c: Brand love has a positive impact on brand engagement.

### Brand Engagement

Customer brand engagement is the state of consumer’s mindset depicting their motivation related to brands characterized with emotional, cognitive, and behavioral activities pertaining to consumer interaction with brands (Hollebeek, 2011). In the academia, the term “engagement” has been utilized in a variety of academic, such as psychology, sociology, political science, and organizational behavior (Brodie et al., 2011). In the literature pertaining to marketing, the terms “consumer engagement,” “customer engagement,” and “brand engagement” have been used interchangeably since 2005 (Brodie et al., 2011). Brand engagement in self-concept is “an individual difference representing consumers’ propensity to include important brands as part of how they view themselves” (Sprott et al., 2009).

Today’s competitive environment where there are a variety of brands available with dynamic technology has communicated to companies that consumers are not mere purchasers of brands, and that they indeed can contribute more to brand development (Brodie et al., 2011; Kumar and Pansari, 2016; Gupta et al., 2018). These opportunities are also available for consumer-to-consumer interactions through different online communities, blogs, and social media platforms (So et al., 2016). More specifically, in today’s technologically advanced context, consumers can not only purchase brands, but they can also impact the brands by sharing their experiences with other consumers and brands using social media. Consumer feedback in this way is important for improving the services of brands (Gupta et al., 2018). Therefore, in current technologically advanced context, keeping customers engaged is paramount for companies to remain competitive (Kumar and Pansari, 2016).

#### Brand Engagement With Customer Delight and Psychological Well-being

Research has documented the importance of consumer brand engagement for a variety of outcomes related to brands as well as consumers such as positive word-of-mouth, consumer delight, customer well-being, brand referrals, and brand loyalty (Junaid et al., 2019; Singh and Srivastava, 2019; Algharabat et al., 2020). The following section outlines possible predictors and consequences of consumer-brand engagement. This discussion is the basis for the development of hypotheses for the study. Numerous researchers have studied the relationship of brand engagement with other constructs. Loureiro et al. (2017) who conducted a study on the millennial generation that uses electronic devices for online interaction and found that online brand experience, brand involvement, and self-brand image congruity are predictor of brand engagement. Other researchers found that brand love is a crucial determinant of brand engagement (Junaid et al., 2019, 2020; Samala and Singh, 2019).

Many researchers have found that brand engagement is a predictor of several outcome variables (Junaid et al., 2019, 2020). Carvalho and Fernandes (2018) suggested that customer brand engagement has a positive relationship with customer word of mouth, customer trust, and customer commitment. In the automobile industry, Adhikari and Panda (2019) found that the engagement of customer brand positively impacted relationship quality and brand loyalty. Furthermore, researchers found that brand engagement is a strong predictor of customer well-being (Junaid et al., 2019, 2020). Anam and Faiz (2016) found that brand engagement, brand attachment, surprise, and customer satisfaction are predictors of customer delight. There is further needed to explore the brand engagement relationship with customer delight and consumer well-being specifically psychological well-being. On the basis of the given literature, these hypotheses are presented in this study:

H3a: Brand engagement has a positive impact on customer delight.
H3b: Brand engagement has a positive impact on psychological well-being.

### Brand Love as Mediator

Brand love has been taken as a mediator in various studies and contexts. Junaid et al. (2020) conducted a study on tourist well-being in which they found a mediating relationship between brand love and perceived value of tourists and their well-being. Khan et al. (2021) also found mediation of brand love between perceived benefits and brand loyalty in the smartphone industry. In another study, Junaid et al. (2020) found brand love as a mediator between participation in co-creation and consumer well-being. Tiwari et al. (2021) found that brand love is strongly predicted by the perceived coolness and that there is also a strong relationship between brand love and well-being of consumers (Junaid et al., 2019) and customer delight. On the basis of the mentioned literature, the following hypotheses are supported:

H5a: Brand love mediates the relationship between brand coolness and customer delight.

H5b: Brand love mediates the relationship between brand coolness and psychological well-being.

### Brand Engagement as Mediator

Various researchers found a mediating role of brand engagement in their studies in different contexts. Kaur et al. (2020) found that customer brand engagement mediates the relationship among brand community identification, rewards, and brand loyalty in the context of virtual brand communities. Junaid et al. (2020) found that brand engagement mediates the relationship between brand love and customer well-being. Lima (2020) found brand coolness as a predictor of brand engagement, and brand engagement as a predictor of consumer delight and customer well-being. Therefore, there is a need to explore the mediating role of brand engagement in the relationship between brand coolness and consumer delight and psychological well-being based on this literature:

H6a: Brand engagement mediates the relationship between brand coolness and customer delight.

H6b: Brand engagement mediates the relationship between brand coolness and psychological well-being.

### Customer Delight

Customer delight has always been a center of interest for both scholars and practitioners (N. Torres et al., 2014). Customer delight is defined as a “profoundly positive emotional state where expectations are exceeded to a surprising degree” (Oliver, 1997). Customer delight refers to enhanced levels of happiness felt by consumers related to their consumption experience (Kumar, 1996). Chandler (1989) argued that customer delight is the level of satisfaction beyond consumer anticipation resulting from a consumption experience. Although customer delight and customer satisfaction are interrelated, some scholars argue that customer delight is a recent phenomenon that needs further investigation as to its relationship with other concepts (Crotts and Magnini, 2011; Ma et al., 2013). Ahrholdt et al. (2016) emphasized to focus on studying customer delight in the service industry to see how service quality improves customer delight beyond customer satisfaction.

Moreover, researchers (Kumar et al., 2001) explored the psychological aspects of customer delight and have considered it as related to the enthusiasm, joy, and thrill displayed by consumers who receive a better service quality. In their investigation on supermarket shoppers, Barnes et al. (2016) found different variables leading to customer delight. Specifically, they found a strong association between joy and surprise and customer delight. Besides proposers of customer delight, some scholars such as Kim et al. (2015) shared different viewpoints. They argued that delight is not as powerful as satisfaction because of its strong impact on customer attitudes and behaviors. Dubey et al. (2020) studied different values (i.e., perceived price, perceived sacrifice, perceived benefits, perceived bargain, brand value, utilitarian value, transactional values, epistemic value, hedonic value, and self-congruity) with customer delight in mobile technology context in India.

Anam and Faiz (2016) studied surprise, brand attachment, customer satisfaction, and customer brand engagement with customer delight and brand loyalty. In their study, they found that customer brand engagement positively affects customer delight. Barnes and Krallman (2019) conducted a great study on customer delight and found antecedents and outcomes of customer delight in different perspectives, i.e., employee perspective, customer perspective, and contextual perspective. They suggested that joy, surprise, arousal, fun, comfort, and expectations are antecedents in customer perspectives, and that loyalty, brand beliefs, impulsive purchase, consciousness, word of mouth, and repurchase intentions are outcome of customer delight. Based on the given literature, this study tries to explore the psychological antecedents (brand love and brand engagement) of customer delight.

### Customer Psychological Well-being

Well-being refers to one’s condition and health. It has been studied in a variety of contexts such as physical, psychological, economic, or social state (Diener, 2009). This has resulted in a variety of definitions attributed to the concept of well-being with references to the context it is being studied such as social and economic well-being, and psychological and consumer well-being (CWB). CWB, like other concepts of well-being lacks a specific definition (Sirgy et al., 2007). In general, CWB implies the well-being of consumers (Lee and Ahn, 2016). More specifically, CWB entails consumption-related aspects of an individual and merits happiness derived out of consumption of goods and services. It also refers to what brands contribute in the life satisfaction of consumers (Grzeskowiak and Sirgy, 2007; Kim et al., 2012). Practically, it refers to a degree at which consumption of brands enhances consumers’ feeling of quality of life (Sirgy et al., 2007).

The CWB concept has emerged in marketing literature very recently. It is rooted in the notion of the role marketing plays in economic and social aspects of consumers’ lives (Sirgy et al., 2007). It has actually furthered the concepts of consumer satisfaction and consumer delight, which are just specific to the performance of brands; however, consumer well-being goes beyond brand features, and it is related to contributions brands make in the improvement of consumers’ quality of life. Greater levels of quality of life achieved out of consumption of brands will reduce ill-being and foster social well-being and overall happiness (Grzeskowiak and Sirgy, 2007). That is why consumer well-being is becoming an end objective for consumers’ consumption-related decisions (Sirgy et al., 2007). Consumer well-being is “a state of flourishing that involves health, happiness, and prosperity.” Kim et al. (2012) conducted a research on consumer-well-being with brand attitude and perceived values (utilitarian values and hedonic values). The results showed that brand attitude and values positively affect consumer well-being.

Ogunmokun et al. (2021) conveyed a study on antecedents of consumer well-being in higher education context. They proposed customer engagement, and the perceived service features were the anterior well-being of consumers. In another study, Junaid et al. (2019) found brand engagement as a strong predictor of customer well-being. They also quantified that brand engagement mediates the relationship between brand love and customer well-being. Customer well-being is not explored well in marketing studies, and there is a need to explore customer well-being, specifically customer psychological well-being (Kemp et al., 2020). On the basis of this discussion, the study seeks to find the antecedents of customer psychological well-being.

### Brand Experience

The “brand experience” concept has thus raised the attention of academics and interpreters. Brand experience has been well-defined as “sensation, feeling, cognition, and behavioral responses evoked by brand-related stimuli that are part of a brand design and identity, packaging, communications, and environments” (Brakus et al., 2009). The experience of the brand indicates the experience of the customer with brand and any of the organization whose brand being purchased and consumed by the customers, the brand also effect on the persons who don’t even consume the brand (Khan and Rahman, 2015). Brands are considered as important resources for companies; therefore, managing consumers’ experience of brands is an important aspect of the marketers’ job. However, focusing on consumers’ benefits accrued out of brands is not sufficient, as consumer experiences have lasting effects on brand progress (Ong et al., 2018). Consumer experiences are translated through different aspects of brands such as aesthetics (environment *per se*), joy (provision of entertainment), education (provision of knowledge and live experience), and escapism (full immersion with experience). Cleff et al. (2014) and Suntikul and Jachna (2016) argued that brands not only provide functional benefits but that they also contribute to consumers’ experience.

Unlike interpersonal relationships, love at first sight is not always applicable in brands; it is an experience that develops affection and love toward a brand (Langner et al., 2016; Prentice et al., 2019). Positive consumer experiences of brands foster consumer engagement with the brands that shape customer behaviors and intention to revisit and repurchase the brands. Thus, marketers need to focus on relevant stimuli to make a consumer experience more positive and memorable, so benefits of consumer engagement may be achieved for a longer period (Ahn and Back, 2018). Other researchers found that brand experience has a positive influence on brand love (Ferreira et al., 2019; Rodrigues and Brandão, 2021; Safeer et al., 2021).

#### Brand Experience as Moderator

Brand experience, unlike attitude toward a brand, is a direct outcome of consumption of a product or a service. This experience may be different for different consumers (Joshi and Garg, 2021). Studies reveal that occasional brand experience cannot result in brand love unless it is repeated and accumulated by previous past positive experiences; however, brand attitude does not require an extensive and repeated experience (Park et al., 2010). Keeping in view this complex relationship of brand experience with brand love and engagement, this study considers that the effect of brand coolness on brand love and brand engagement will be different on different levels of brand experience. Previous studies have also tested the moderating role of brand experience. Joshi and Garg (2021) also investigated brand experience as a moderator among brand image, brand satisfaction, and brand love. Keeping in view the limited evidence of moderation of brand experience, this study intends to extend the literature by testing the moderating role of brand experience between brand coolness and brand love, and brand coolness and brand engagement:

H7a: Brand experience moderates the relationship between brand coolness and brand love.

H7a: Brand experience moderates the relationship between brand coolness and brand engagement.

### Theoretical Framework

**FIGURE 1:**
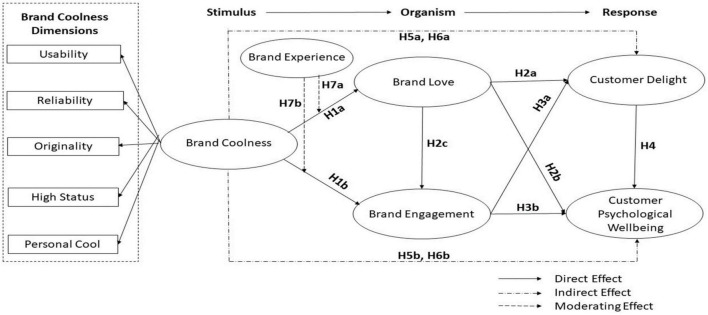
Theoretical framework.

Based on the theory of the attribution of brand and model of stimulus-organism-response (SOR), here is the theoretical framework that has been constructed in our research (see Figure 1).

## Materials and Methods

### Sample and Procedure

Since a survey is conducted to analyze correlations between constructs and to measure behaviors, a survey utilizing a quantitative method was performed to collect primary data from respondents. The survey helped the research to collect data quickly and was easy to administer. The survey method is cost-effective, and researchers can collect large amounts of data. The information was gathered from young smart device users in Pakistan’s major cities (i.e., Islamabad, Karachi, Rawalpindi, Lahore, and Multan) through a survey conducted at malls, shopping centers, bus stops, and universities. To establish content validity and refine the instrument, a focus group was conducted with 3 smartphone users and three scholars (with a specialty in marketing). The questionnaire was pre-tested before it was finalized to ensure its validity and reliability. The pilot study included 70 smart gadget users, and they were later excluded from the final data analysis. Most of the measuring scales were shown to be valid in the pilot study. Scales that were previously unfavorable were modified. Purposive sampling was conducted to find people who shared certain qualities that were related to the research’s goals. We underlined that there are no right or incorrect answers to reduce social desirability bias and to maintain secrecy and anonymity. The survey was conducted in the English language for a host of reasons. First, based on the researchers’ anecdotal experience, a considerable number of target audience of smart gadget users in Pakistan is comprised of urban residents that are well-educated and employed in multinational companies that require English language proficiency. Hence, communication in English is not a major concern for such users. Moreover, previous research has established that English is widely spoken by Pakistanis (Islam et al., 2019). We sent out 1,800 survey forms, from which responses were received from 1,213 people. After removal of missing values, the final data set had 1,178 replies, resulting in a response rate of 65.44%. There were too many items in the survey, and we approached the respondents in malls and shopping centers. There were respondents who did not complete the survey fully and questionnaires that were invalidated. Because of these reasons, the response rate was low. The demographic information of the participants is shown in Table 1.

**TABLE 1:** Sample characteristics.

Demographic	Category	Percentage (Frequency)
Gender	Male	53.1 (626)
	Female	46.9 (552)
Age (In years)	Less than 18	7.4 (87)
	18–22 years	40.2 (473)
	23–27 years	34.9 (409)
	28–32 years	17.5 (204)
Education	High School	11.9 (141)
	Professional degree/vocational school	17.1 (201)
	Bachelors	37.9 (447)
	Masters	30.6 (360)
	Doctorate	2.5 (29)

A total of 1,178 people took part in the study, with 626 (53.1%) of them being male smart gadget users and 552 (46.9%) being female smart gadget users. Since the information was gathered from smart device users, the level of education showed that 17.9 percent of the respondents have only an intermediate education. Table 1 shows that 37.9 percent of the participants have a bachelor’s s, that 30.6 percent have a master’s degree, and that just 2.5 percent have a doctoral degree. To check for normality, skewness and kurtosis were evaluated. The resultant values were within the range of −1 to +1 and −3 to +3, respectively.

### Measures

Using existing scales, all the constructs were modified and conceptualized. The construct items were derived from a variety of measurement scales, and information was taken on a five-point Likert scale. Usability, reliability, uniqueness, high prestige, and personal cool are the five dimensions of brand coolness. Table 2 summarizes all the measures.

**TABLE 2:** Measurement scales with outer loadings.

Constructs	Code	Statements	Mean, (S.D.)	Loadings
Usability (Raptis et al., 2017)	usb1	My smartphone is simple to use.	3.20, (1.15)	0.80
	usb2	My smartphone is easy to operate	3.35, (1.18)	0.87
	usb3	My smartphone is easy to learn	3.42, (1.14)	0.88
	usb4	My smartphone is easy to use	3.28, (1.15)	0.86
Reliability (Tiwari et al., 2021)	rea1	My smartphone provides the services as promised.	3.34, (1.24)	0.84
	rea2	My smartphone performs tasks right every time.	3.54, (1.19)	0.89
	rea3	My smartphone rarely hangs/stops working.	3.49, (1.14)	0.90
	rea4	My smartphone is dependable in handling	3.48, (1.18)	0.87
Originality (Kim et al., 2015)	org1	This smartphone is original	3.68, (1.26)	0.88
	org2	This smartphone is unique	3.50, (1.17)	0.87
	org3	This smartphone stand apart from similar products	3.65, (1.18)	0.87
High Status (Loureiro et al., 2020)	hst1	This luxury fashion brand is chic.	3.23, (1.21)	0.81
	hst2	This luxury fashion brand is glamorous.	3.17, (1.10)	0.83
	hst3	This luxury fashion brand is sophisticated.	2.56, (1.28)	0.69
	hst4	This luxury fashion brand is ritzy	2.63, (1.25)	0.73
	hst5	This luxury fashion brand is attractive	3.22, (1.18)	0.77
Personal Cool (Runyan et al., 2013)	pcl1	A “cool” article of clothing fits my personality	3.21, (1.13)	0.84
	pcl2	A “cool” article of clothing boosts my confidence	3.20, (1.10)	0.85
	pcl3	A “cool” article of clothing fits my self-identity	3.19, (1.10)	0.85
	pcl4	A “cool” article of clothing boosts my self-esteem	3.32, (1.14)	0.83
	pcl5	A “cool” article of clothing fits my style	3.25, (1.15)	0.83
	pcl6	A “cool” article of clothing contributes to my individuality	3.29, (1.12)	0.84
Brand Love (Carroll and Ahuvia, 2006)	brl1	This is a wonderful brand.	3.16, (1.14)	0.80
	brl2	This brand makes me feel good.	3.41, (1.12)	0.87
	brl3	I love this brand!	3.46, (1.14)	0.88
	brl4	This brand is totally awesome.	3.44, (1.14)	0.86
	brl5	This brand makes me very happy.	3.40, (1.14)	0.85
	*brl6*	This brand is a pure delight	3.37, (1.12)	*0.59*
Brand Engagement (Xi and Hamari, 2021)	beg1	I love talking and using products of the brand with my friends	3.39, (1.15)	0.75
	beg2	I enjoy talking and using products of the brand more when I am with others	3.46, (1.10)	0.78
	beg3	Talking and using products of the brand are more fun when other people around me do it too	3.24, (1.19)	0.84
	beg4	I feel good about sharing my experiences with the products of the brand with others	3.11, (1.19)	0.83
	beg5	I feel fellowship with other people who use the products of the brand	3.06, (1.21)	0.82
	beg6	I like recommending the products of the brand to others	3.10, (1.23)	0.81
Customer Delight	cde1	I was delighted by this experience	3.15, (1.23)	0.83
	cde2	It was a thrilling experience	3.33, (1.13)	0.87
	cde3	It was an exhilarating experience	3.26, (1.10)	0.88
	cde4	I was pleased with this experience	3.27, (1.13)	0.87
Customer Psychological Well-being (Grzeskowiak and Sirgy, 2007)	cwb1	This smartphone plays a very important role in my social well-being.	3.27, (1.17)	0.84
	cwb2	This smartphone plays an important role in my leisure well-being.	3.39, (1.10)	0.84
	cwb3	This smartphone plays an important role in enhancing the quality of my university life.	3.47, (1.13)	0.85
	cwb4	This smartphone satisfies my overall needs	3.26, (1.21)	0.83
Brand Experience (Khan et al., 2021)	bex1	I have strong emotions for Brand X	3.18, (1.25)	0.85
	bex2	I engage in physical actions and behaviors when I experience Brand X.	3.44, (1.19)	0.89
	bex3	Brand X results in lively experiences.	3.50, (1.21)	0.90
	bex4	Brand X is willing or likely to take practical action to deal with a problem or situation	3.37, (1.19)	0.89
	bex5	Brand X stimulates my curiosity and problem-solving	3.40, (1.23)	0.87

### Data Analysis

To investigate the structural model, we conducted structural regression modeling by partial least square (PLS) estimation. PLS is quite popular and widely used because of its ability to integrate linear regression with confirmatory factor analysis. PLS is also more accurate than covariance-based structural equation modeling in finding actual paths and not detecting non-existent paths (Goodhue et al., 2012). This method is meant for creation and examination of complex interactions between numerous variables. This technique is also useful for testing hypotheses and determining the relationship between various variables. It also assists in the analysis of latent variable causal relationships. PLS is an excellent technique for doing confirmatory factor analysis (CFA) and regression when testing a measurement and structural model (Hair et al., 2017). Smart-PLS is a cutting-edge program that was used to assess the measurement and structural model. We utilized Smart-PLS 3.3 to perform the PLS analysis in this study.

## Results

### Common Method Bias

Common method variance (CMV) was found to be a source of concern in our study. As a result, we implemented a number of procedures to mitigate and minimize its impact on the outcomes. We used the techniques recommended by Podsakoff et al. (2003) to decrease CMV. The anonymity of respondents is protected first and foremost. Second, we integrate things from the available literature to eliminate item uncertainty. Third, the survey’s items were arranged in a random order. Consequently, we carried out Harman’s single factor test to see if the results in this study had a CMB that is in agreement with recent investigations (Talwar et al., 2020). The findings showed that a single factor could only describe 42.66% of the variance, which was significantly less than the cut-off value of 50% (Podsakoff et al., 2012). There was no CMB in the data; therefore, they were suitable for statistical analysis.

### Measurement Model

Outer loads are considered first in a measurement model. Evaluating the observed constructs and their related items improves the significance of a measurement model. For this reason, each item’s outer loading is evaluated. Any item having loading less than 0.5 is deleted based on the criteria (Hair et al., 2019). The next stage is to analyze the reliability and validity of all the constructs after a thorough assessment of loadings. Composite reliability and Cronbach alpha are two important criteria to consider while evaluating reliability (internal consistency). The final Cronbach alpha values varied from 0.88 to 0.95 and showed a high level of reliability (>0.7) (Hair et al., 2019). Composite reliability is the next measure of internal consistency. It was calculated using the outer loadings of all variables. The resulting composite reliability ratings varied from 0.91 to 0.95, showing good consistency (i.e., a number greater than 0.7) (Hair et al., 2019). Convergent validity can be used to investigate the relationship between all items in a concept. Average extracted variance (AVE) is used to assess the convergent validity of variables. The convergent validity of the variables ranged from 0.51 to 0.78, suggesting an excellent convergent validity (>0.5) (Hair et al., 2019). Table 3 summarizes the findings.

**TABLE 3:** Internal consistency and discriminant validity (heterotrait-monotrait, HTMT, and ratio) evaluation.

	Cronbach α	CR	AVE	BC	BL	BE	CD	WB	BX
BC	0.95	0.95	0.51						
BL	0.90	0.93	0.72	0.79					
BE	0.89	0.91	0.65	0.72	0.77				
CD	0.88	0.92	0.74	0.70	0.73	0.93			
PWB	0.91	0.93	0.70	0.83	0.82	0.74	0.71		
BX	0.93	0.94	0.78	0.78	0.82	0.76	0.74	0.80	

*BC, brand coolness; BL, brand love; BE, brand engagement; CD, customer delight; PWB, psychological well-being; BX, brand experience.*

Finally, the study variables’ discriminant validity is assessed. This is performed by determining discriminant validity using the heterotrait-monotrait (HTMT) technique. The value of HTMT should not exceed 0.95 (Hair et al., 2019). Table 3 summarizes the findings and proves that discriminant validity exists.

### Structural Model

For all the constructs, variance inflation factor analysis was conducted to determine multi-collinearity. The result of VIF was less than 3.3, which is recommended by Hair et al. (2017), and showed that there was no issue with multi-collinearity. The results are summarized in Table 4.

**TABLE 4:** Multi-collinearity evaluation.

	BL	BE	CD	PWB
BC	1.00	2.83		
BL		2.98	2.03	2.12
BE			2.03	3.23
CD				3.23
BX		3.03		

*BC, brand coolness; BL, brand love; BE, brand engagement; CD, customer delight; PWB, psychological well-being; BX, brand experience.*

**FIGURE 2:**
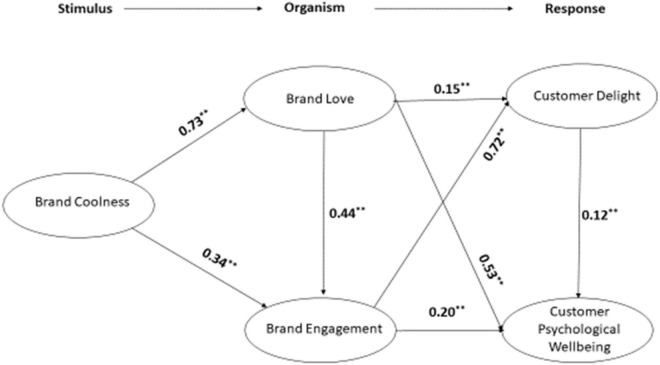
Structural model. **Means p value < 0.00.

The hypotheses are then tested in the second stage. All of the hypotheses were confirmed by the findings; hypothesis 1a (brand coolness influences brand love) is confirmed (β = 0.73^***^). Hypothesis 1b (brand coolness influences brand engagement) is confirmed (β = 0.34^***^). Hypothesis 2a (brand love influences customer delight) is confirmed (β = 0.15^***^). Hypothesis 2b (brand love influences customer psychological well-being) is confirmed (β = 0.53^***^). Hypothesis 2c (brand love influences brand engagement) is accepted (β = 0.44^***^). Hypothesis 3a (brand engagement influences customer delight) is accepted (β = 0.72^***^). Hypothesis 3b (brand engagement influences customer psychological well-being) is accepted (β = 0.2^***^). Hypothesis 4 (customer delight influences customer psychological well-being) is confirmed (β = 0.12^***^) (see Figure 2). The *R*^2^ values are 0.57, 0.58, 0.70, and 0.63 for brand love, brand engagement, customer delight, and psychological well-being, respectively. The Q^2^ (blindfolding) values are 0.39, 0.34, 0.51, and 0.43 for brand love, brand engagement, customer delight, and psychological well-being, respectively and are greater than 0 (Hair et al., 2019) (refer to Appendix).

Similarly, the mediating effect hypotheses, H5a, H5b, H6a, and H6b, are accepted (β = 0.44, *p* < 0.00; β = 0.44, *p* < 0; β = 0.44, *p* < 0; β = 0.44, *p* < 0, respectively) (refer to Table 5).

**TABLE 5:** Hypotheses assessment.

Hypothesis	Path	Estimate	S.D.		*T*-value	*P-value*
H_1a_	BC→BL	0.73	0.01		44.51	***
H_1b_	BC→BE	0.34	0.03		8.39	***
H_2a_	BL→CD	0.15	0.02		6.13	***
H_2b_	BL→PWB	0.53	0.03		17.67	***
H_2c_	BL→BE	0.44	0.03		11.98	***
H_3a_	BE→CD	0.72	0.02		27.48	***
H_3b_	BE→PWB	0.20	0.04		4.42	***
H_4_	CD→PWB	0.12	0.03		3.01	***
H_5a_	BC→BL→CD	0.11		[LCL = 0.08, UCL = 0.14]		***
H_5b_	BC→BL→PWB	0.39		[LCL = 0.35, UCL = 0.43]		***
H_6b_	BC→BE→CD	0.24		[LCL = 0.20, UCL = 0.29]		***
H_6b_	BC→BE→PWB	0.07		[LCL = 0.04, UCL = 0.10]		***

***p < 0.001, BC, brand coolness; BL, brand love; BE, brand engagement; CD, customer delight; PWB, psychological well-being; BX, brand experience.

**FIGURE 3:**
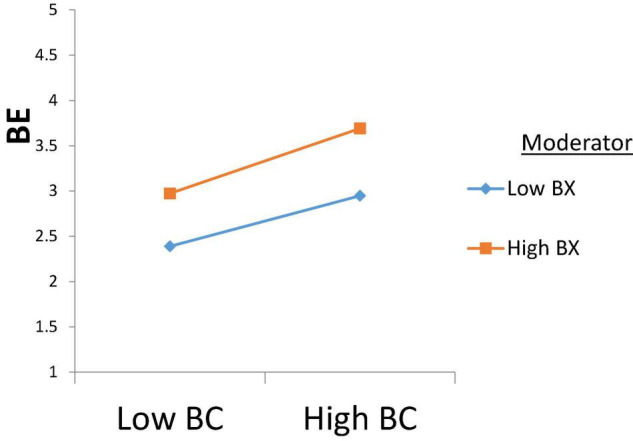
Graphical representation of moderating brand experience.

Furthermore, a hypothesis related to moderating effect, H7a, is not accepted, as *p* > 0.05. However, H7b is accepted as interaction term coefficient β = 0.04, *p* < 0.02, which illustrates that brand experience strengthens the association between brand coolness and brand engagement. The result are depicted in Figure 3, which shows that brand experience strengthens the relationship between brand coolness and brand engagement.

## Discussion

The major hypotheses included the relationship of brand coolness with brand love and brand engagement. Brand love and brand engagement were hypothesized to mediate the relationship of brand coolness with customer delight and customer psychological well-being. Brand experience was modeled as a boundary condition to moderate the link between brand coolness and brand love, and brand coolness and brand engagement. As the link between brand coolness and brand love, and brand love and brand engagement was already tested by some studies in the extant literature, other linkages such as how brand coolness and brand love contribute to customer delight and well-being were not tested earlier.

The first hypothesis was concerned about the impact of brand coolness on brand love. It was supported by the results of this study. This finding suggests that brands that are perceived to be cool, such as those having features like reliability, dependability, usability, uniqueness, and being cool, contribute to consumers’ positive emotions such as brand love. Although empirical research regarding the link between brand coolness and brand love is scarce and at an emerging stage; limited previous research, however, has supported the notion regarding the role of consumers’ perception of coolness of brands in development of intense positive emotions such as brand love and passion. For example, Tiwari et al. (2021), in a sample drawn from smart phone users in India, found that when consumers perceive that their technology products are highly reliable, useable, rebellious, innovative, attractive, and desirable, they feel like falling in love with brands. This bond becomes stronger and initiates other positive emotions. This finding is further supported by other recent studies (Ashfaq et al., 2021). There may not be a lot of research on the relationship between brand coolness and brand love; consequently, this research has a useful addition.

The second hypothesis was concerned with the impact of brand coolness on brand engagement. Brand engagement refers to more active involvement of consumers with brands beyond merely having positive feelings such as brand love. Despite the importance of brand engagement, empirical findings on the link between brand coolness and brand engagement are lacking. However, one research study on beauty brands found a positive association between brand coolness and brand engagement (Serras, 2020). In light of the brand attribution theory, the relationship between brand coolness and brand engagement is important. As coolness perception may be attributed and regarded in terms of enhanced quality and functionality of products, thus consumers having higher levels of coolness perception will be willing to engage with brands and communicate with others about their positive engagement (Bıçakcıoğlu et al., 2018; Rodrigues and Brandão, 2021). Lima (2020) also reported a positive relationship between brand coolness and brand engagement in a sample of social media users. This finding supports the notion that coolness perceptions ignite positive emotions toward brands in consumers (Blanco, 2020). One reason to this phenomenon is that the coolness feature is attributed to the higher status and prestige of a brand. Having prestigious brands in possession develops positive emotions toward brands (Dar-Nimrod et al., 2018).

H2a was concerned about the relationship between brand love and customer delight. Although brand love is a relatively newer construct in the marketing literature (Carroll and Ahuvia, 2006), scholars have been more interested to know about the romantic relationship between consumers and brands. Roberts (2005) conceptualized brand love based on interpersonal love. They theorized that brand love is based on sensational attraction, closeness, devotion, compassion, desire, and aspirations. Carroll and Ahuvia (2006) also argued that brand love is similar to interpersonal love and results in good feelings, happiness, and delight. Roy et al. (2013), in their conceptual article, discussed various outcomes of band love including romanticism, customer satisfaction, and consumer delight. Similarly, H2b was concerned about the impact of brand love on psychological well-being. The results are in support of the hypothesis and imply that as consumers feel themselves to be in a romantic relationship with the brands, it makes them feel better, well-off, and happy. Such finding is consistent with empirical research. Junaid et al. (2019) found a positive association between brand love and consumer well-being. As consumer well-being reflects consumers’ perception of quality of life and feeling of happiness, brand love can best predict well-being, as brand love is the result of consistent satisfaction derived from positive experiences and likeness of a brand.

H2c was also concerned about the association between brand engagement and brand love. Empirical findings confirmed the brand love, as a positive emotion, positively impacts customers’ engagement with brands. This finding is further supported by previous studies such as Junaid et al. (2019), who found that brand love predicts brand engagement. The literature suggests intense positive feelings toward brands such as brand love develop more active participation of consumers with brands such as brand engagement, which is characterized by vigor, dedication, and absorption (1). Therefore, if brands are able to generate positive feelings of customers toward them, consumers may engage more actively with brands (Junaid et al., 2020; Joshi and Garg, 2021).

H3a was about the relationship between brand engagement and customer delight. The findings supported this hypothesis, suggesting that when consumers are engaged with their loved brands, they feel happiness and satisfaction with the brands. The higher the levels of engagement, the higher consumer delight will be (Choi and Hwang, 2019). The authors suggest that positive consumer experiences with a brand will enhance their engagement with the brand, which will further foster their delight and satisfaction (Joshi and Garg, 2021). H3b found a positive impact of brand engagement on customer psychological well-being. This finding again implies that when consumers feel enthusiasm about a brand, they tend to feel better in life while remaining in a consumption relationship with the brand. Such tendency to feel belonging to the brand creates consumer feelings of happiness and thrill.

H4 was about the relationship between customer delight and well-being. The findings supported this link as well, implying that consumers are happy with the brand experience because they are happy with their life and have higher perceptions of quality of life (Netemeyer et al., 2004). Moreover, the finding also implies that when consumers feel delighted and thrilled in using certain brands, they feel themselves to be a very sensible person and capable of decision-making as their decisions lead to obtain the brand that provides them better experience as per their exceptions (Lee and Park, 2019).

H5a was about the relationship between the mediating role of brand coolness and consumer delight. The results supported this hypothesis. According to the SOR model, certain contextual factors act as stimuli and influence the organism to result in a response. In this study, brand coolness, as a consumer-level perception of brands’ various features, is a strong stimulus to stimulate positive emotions such as brand love. Brand love, as an organism, results in responses such as customer delight. Zhu et al. (2019) also used the SOR model in their study and tested brand love as an organism in the model. The role of brand love as a mediator between brand coolness and customer delight is new in the literature; however, past research found that brand love mediated the link between brand experience and consumer satisfaction (Ferreira et al., 2019). H5b was related to mediation of brand engagement between brand coolness and brand delight. This relationship was also substantiated by the findings of this study, implying that when more consumers engage with brands, they will form a positive image of the brands and consider the brands as cool, reliable, dependable, and useable. These feelings will also stimulate consumers to form a perception of delight and satisfaction (Ferreira et al., 2019).

H6a was also concerned about the mediating role of brand love between brand coolness and consumer delight. This study has also supported this hypothesis, implying that brand coolness is such a powerful stimulus that it will encourage consumers to engage with a brand. Brand engagement will later result in satisfied and delightful consumers. Similarly, H6b was about the mediating role of brand engagement between brand coolness and consumer well-being. The findings also corroborated this relationship. This finding implies that customers engaged with brands not only feel satisfied but that their feeling about their quality of life also increases positively (Junaid et al., 2020). The research has acknowledged the association between brand engagement and satisfaction (Fernandes and Moreira, 2019), and this study has supported the mediating role of brand engagement between brand coolness and well-being. Research on HRM studies also supports the idea that engaged employees show greater levels of psychological well-being (Robledo et al., 2019). There are also other studies that support the relationship between brand engagement and consumer well-being (Kumar and Nayak, 2019).

H7a was related to the moderating role of brand experience between brand coolness and brand love. This relationship was found to be insignificant. H7b was related to the moderating role of brand experience in the relationship between brand coolness and brand engagement. The findings supported this link. As stated earlier, the moderating hypothesis between coolness and brand love was not supported. However, the relationship between brand coolness and engagement was supported. This difference may be attributed to the difference between love and engagement, as love reflects one’s admiration to and likeness of a target, whereas engagement is an action-oriented construct that reflects one’s intention to act in a certain way (Kumar et al., 2019). Brand experience is also an active construct that is based on a solid experience. Therefore, the coolness of brand may stimulate one’s affective states such as love and likelihood (Erhan et al., 2020), which may not be influenced by experience. However, an engagement is based on the level of consumer experience; the more consumers experience a brand, the greater their engagement with the brand will be.

### Conclusion

The major conclusions of this study are related to evidence of the relationship among brand coolness, brand love, and engagement. Brand coolness also fosters customer well-being and customer delight. The findings are important in the sense that they reflect the positive sides of brand coolness described in terms of brand reliability, usability, prestige, and personal coolness. The conclusions imply that coolness of brand can foster customer delight by increasing brand love and engagement. Moreover, these variables are also important for the well-being of consumers. Happy consumers are indeed a great asset for organizations. This study has a variety of inferences emerging out of it. For example, this study suggests that if brands are able to develop coolness perceptions such that customers perceive brands to be useful, unique, and rebellious, they can sense that the brands are cool; thus, they can fall in love with the brands. Their engagement with brands can also increase, which will further make them happy, delighted, and psychologically well-off. Moreover, brands can make consumers more engaging by increasing the reliability of their brands and improving the services. Happy consumers are expected to share their positive experience with others and remain loyal to brands. From a theoretical perspective, this study used the SOR model by integrating it with the brand attribution theory. The basic premise of the SOR model is that the certain conditions in the environment function as stimuli to have an impact on consumers’ internal state of mind (organism) and develop consumer behavior. From an attribution theory perspective, it is assumed that people tend to reflect on their past experiences to guide their future decisions and behaviors. By integrating both theories in the brand management literature, this study assumed that when consumers find a certain brand higher in terms of brand coolness (stimulus), it will stimulate brand love and brand engagement (organism), which will in turn result in customer delight and customer well-being (response).

### Theoretical and Practical Implications

This study offers important implications for academicians, practitioners, and policy-makers. This study used the SOR theory and the brand attribution theory to understand the basic mechanism that prevails in the external aspects of brands that stimulate a response. More specifically, this study found that consumer perceptions of brand coolness generate positive emotions and active engagements, which are important for customer delight and psychological well-being. Both the predictors and the outcomes of this study are important contributions in the literature such that customer psychological well-being is now an important aspect of the marketing literature, which is getting popular. The typical marketing literature, focused on customer satisfaction, has progressed further to address more macro-level variables like customer psychological well-being and customer delight. The outcomes have some lasting effects and thus can be a catalyst for further benefits of brands and organizations. This study offers various academic insights. For marketers, this study is a strong proponent of focusing on features that are considered to be contributing to coolness perceptions. Again, this literature extends beyond the functional benefits offered by brands; it focuses on emotion stimulant features like brand coolness that create a strong bond between consumers and brands. For marketers that emphasize on brand consumer interactions, the findings of this study are a strong example to follow so as to achieve certain durable outcomes.

This research intended to contribute to the literature of brand coolness. More specifically, it aimed to know how brand coolness contributes to the development of customer delight and customer well-being by enhancement of positive outcomes such as brand love and brand engagement. For doing this, this study utilized the SOR model and integrated it with the brand attribution theory. Brand coolness was measured in terms of its latent dimensions such as usability, originality, reliability, greater status, and personal coolness, and consumer engagement with and love for brands resulting in consumer delight and well-being. This study is significant for academicians and practitioners in the sense that most of the brand management literature has traditionally been focused on consumer satisfaction and delight, but at present times the role of marketing is being acknowledged to develop consumer well-being and happiness (Alexander et al., 2021). This study has responded to such calls and provided a robust model to develop consumer psychological well-being by increasing brand coolness and fostering positive responses in consumers. Furthermore, this study has introduced some new linkages and thus has offered greater contributions for academicians and policy-makers.

### Limitations and Future Recommendations

The major limitations of this study are methodological in nature. The cross-sectional design of this study precludes causality. Recent research emphasizes cross-sectional design. Future research may use longitudinal design with proper time intervals to see how the predictors of this model translate to distant outcomes. This study has used the SOR model and the brand attribution theory to study the impact of brand coolness as a stimulus to emotional and behavioral outcomes. Regardless of the sophistication of the SOR model, the phenomenon of brand coolness perceptions may be understood through the use of self-categorization theory (Trepte and Loy, 2017), which proposes that people, based on their prestigious belongings, compare and categorize themselves to show their attachment and linkage with particular brands or groups. This attachment may ignite certain positive emotions reflecting passion and love and may translate further into delight and well-being. Recognizing this promising possibility, it is recommended that future research should use this theory to test the model of this study.

## Data Availability Statement

The raw data supporting the conclusions of this article will be made available by the authors, without undue reservation.

## Ethics Statement

Ethical review and approval was not required for this study on human participants in accordance with the local legislation and institutional requirements. The patients/participants provided their written informed consent to participate in this study.

## Author Contributions

All authors listed have made a substantial, direct, and intellectual contribution to the work, and approved it for publication.

## Conflict of Interest

AS was employed by AM MCR Ltd., United Kingdom. The remaining authors declare that the research was conducted in the absence of any commercial or financial relationships that could be construed as a potential conflict of interest.

## Publisher’s Note

All claims expressed in this article are solely those of the authors and do not necessarily represent those of their affiliated organizations, or those of the publisher, the editors and the reviewers. Any product that may be evaluated in this article, or claim that may be made by its manufacturer, is not guaranteed or endorsed by the publisher.

## Figures and Tables

**FIGURE A1:**
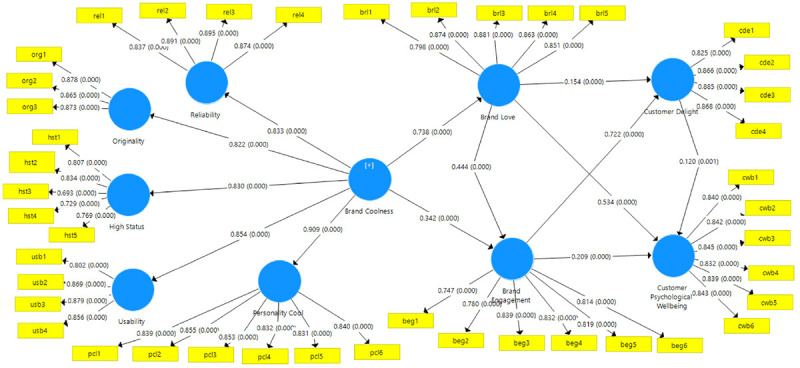
Structural model.
